# Participation in cardiovascular screening consultations, the who, when and why - A cohort study on patients with rheumatoid arthritis

**DOI:** 10.1186/s41927-024-00378-7

**Published:** 2024-02-22

**Authors:** Julie Katrine Karstensen, Ann Bremander, Jeanette Reffstrup Christensen, Jette Primdahl

**Affiliations:** 1 Department of Regional Health Research, University of Southern Denmark, Odense, Denmark; 2 Danish Hospital for Rheumatic Diseases, University Hospital of Southern Denmark, Sønderborg, Denmark; 3 The DANBIO Registry, Rigshospitalet, Glostrup, Denmark; 4 Spenshult Research and Development Centre, Halmstad, Sweden; 5 Section of Rheumatology, Department of Clinical Sciences Lund, Lund University, Lund, Sweden; 6 Research Unit of General Practice, Department of Public Health, University of Southern Denmark, Odense, Denmark; 7 Research Unit for General Practice, Aarhus University, Aarhus, Denmark; 8 DRIVEN - Danish Centre for Motivational and Behaviour Science, Department of Sports Science and Clinical Biomechanics, University of Southern Denmark, Odense, Denmark; 9 Sygehus Sønderjylland, University Hospital of Southern Denmark, Aabenraa, Denmark

**Keywords:** Health behaviour, Cardiovascular risk assessment, Cardiovascular risk management, Rheumatoid arthritis

## Abstract

**Background:**

In accordance with the EULAR recommendations, the Danish Hospital for Rheumatic Diseases have systematically invited patients with rheumatoid arthritis (RA) to cardiovascular (CV) risk assessment since 2011. Patients with high risk are invited to a follow-up screening after one year. To optimize the screening and tailor it to individual needs, information about who accepts vs. declines follow-up is needed. Thus, the aim of this study was to explore participation in systematic CV risk assessment among patients with RA. Furthermore, to explore differences between patients with low vs. high risk, and between patients with high risk who accept vs. decline follow-up.

**Methods:**

Data from 2,222 outpatients with RA in the period 2011-2021 were retrieved, and of these 1,522 were under 75 years and eligible to be invited. To assess the 10-year risk for CV death, the modified Systematic Coronary Risk Evaluation (mSCORE), derived by multiplying the SCORE by 1.5, was used. Logistic regression analyses were used to explore differences in CV risk factors (triglycerides, HbA1c, lifestyle factors) and measures of disease impact (pain, fatigue, patient global assessment, HAQ, EQ-5D-5L) between patients with low vs. high risk. Differences between high risk patients who accepted vs. declined follow-up were analysed using Wilcoxon rank sum test and chi-squared test for groups.

**Results:**

One thousand one hundred forty-nine received a CV screening invitation and 91 declined participation. Patients with high risk had significantly longer disease duration (OR; 95 CI) (1.017; 1.002-1.032), higher levels of triglycerides (1.834; 1.475-2.280), HbA1C (1.046; 1.020-1.070), pain (1.006; 1.001-1.012), and HAQ-score (1.305; 1.057-1.612) compared to patients with low risk and they more often declined follow-up (*43% vs. 28%, p* < *0.001*). Those who declined a follow-up invitation were older (*p* = *0.016*) and had shorter disease duration (*p* = *0.006*) compared to those who accepted follow-up.

**Conclusion:**

A first CV screening consultation was accepted by most patients with RA, while only every other patient with high to very high CV risk adhered to a follow-up screening consultation. Neither measures of disease impact nor lifestyle factors were associated with adherence. Further studies are needed to explore the patients' motivation, barriers and facilitators for adherence or non-adherence to a follow-up consultation.

## Background

Patients with rheumatoid arthritis (RA) have a double risk of developing a cardiovascular disease (CVD) compared to the general population [1, 2]. Accordingly, international guidelines from the European Alliance of Associations for Rheumatology (EULAR) endorse structured cardiovascular (CV) risk management for patients with RA [3]. To assess the ten-year risk for CV death in patients with RA, the use of Systematic Coronary Risk Evaluation (SCORE) is recommended [4]. EULAR recommends that the SCORE is multiplied with 1.5 to reach a modified SCORE (mSCORE) for a more accurate risk prediction [3, 4].

Information about the benefits of smoking cessation, regular physical activity and a healthy diet is also emphasised in the EULAR recommendations for CV risk management [3]. In addition, recommendations from the Danish Society for Rheumatology recommends patients with RA to adhere to the national guidelines on alcohol consumption [5]. EULAR recommendations for lifestyle improvements also highlight the importance of informing patients about the consequences of excess alcohol consumption [6]. The combination of two or more unhealthy lifestyle habits increases the risk for CVD in the general population [7, 8]. However, SCORE does not take level of physical activity, overweight and excessive alcohol consumption into account when assessing the risk for CV death [3], although the level of physical activity is as important a risk factor as smoking [9]. In a previous study, we found that every second patient with RA had two or more unhealthy lifestyle factors [10]. We thus need to discuss the combined number of unhealthy lifestyle factors during the screening consultation. Earlier studies have shown that people with RA find it challenging in agreeing to participate in CV screening and follow-up on lifestyle changes [11, 12].

The Danish Hospital for Rheumatic diseases developed and implemented systematic CV screening consultations in 2011, for all outpatients with RA, based on the EULAR recommendations and national guidelines [13]. The extent of participation in a screening consultation has not yet been explored. Furthermore, there is a need for an improved understanding of whether CV risk factors not included in SCORE, differ in relation to the patient’s mSCORE. To be able to improve the screening consultations, it is also important to learn more about patients with high to very high risk of CV death and whether they adhere to a follow-up CV screening consultation. Thus, the aims of this study were: I) to explore participation in CV screening consultations in patients with RA and CV risk among the participants, II) to explore differences in CV risk factors not included in the SCORE for patients with low to moderate vs. high to very high risk mSCORE, and III) to explore differences between patients with high to very high mSCORE who accept vs. decline participation in a follow-up screening consultation.

## Methods

Data in this register based study were retrieved from all outpatients with RA connected to the Danish Hospital for Rheumatic diseases in the period 2011-2021 registered in DANBIO, a national clinical registry for patients with inflammatory arthritis [14].

### The CV screening consultations

Blood sugar (fasting glucose in the first year and subsequently long-term blood sugar, HbA1C), triglycerides, total cholesterol, high density lipoprotein (HDL) cholesterol and low-density lipoprotein (LDL) cholesterol levels are checked before each screening consultation. During the consultation, blood pressure, height, weight, and waist circumference are measured, and body mass index (BMI) is calculated [13]. Each patient’s risk SCORE is calculated based on age, gender, smoking habits, systolic blood pressure and total cholesterol/HDL-cholesterol ratio [4] and the SCORE is multiplied by 1.5 to reach the mSCORE [3]. The CV screening consultation is performed by a trained rheumatology nurse at the outpatient clinic at the hospital. During the consultation, the nurse enters into a dialogue with the patient about lifestyle factors (diet, smoking, alcohol use and level of physical activity), what fits into the patient’s everyday life, what is important for the patient and motivation for change using elements from motivational interviewing [13, 15]. In accordance with the EULAR recommendations for CV risk management [3], all patients who are below 75 years old are invited to a screening consultation and patients under the age of 70 years are invited to a follow-up screening consultation. Patients with high to very high risk (mSCORE ≥ 5%), or with known CVD or Diabetes Mellitus (DM), are invited to a follow-up screening consultation after one year [3, 4, 13]. Patients with low to moderate risk (mSCORE < 5%) are invited to a follow-up screening consultation every second or third year depending on whether they have modifiable risk factors [13]. The patients’ general practitioner (GP) is informed electronically if a patient declines an invitation to CV screening or do not show up.

### Variables

#### Medical treatment

Medical treatment was self-reported by the patients and divided into four categories: no disease-modifying antirheumatic drug (DMARD) or corticosteroids (CS), conventional DMARD (with or without CS), biological DMARD (with or without CS), and CS only.

#### Lifestyle factors

Smoking, alcohol consumption, and level of physical activity were self-reported and validated by the nurses through dialogue with the patients during the consultations. In addition, we included waist circumference and BMI. All lifestyle factors were categorised as healthy vs. unhealthy based on international and national recommendations.

Patients’ smoking habits were reported as never, previous or present smoker. Report of present smoking was categorised as an unhealthy lifestyle factor. Alcohol consumption was reported as number of units per week. At the time of data collection for this study, the national recommended limits for harmful drinking in Denmark were max. 7 units per week for women and 14 for men [13].

Physical activity level was self-reported as number of days with moderate-intensity physical activity of at least 30 min. The categories were five or more days a week, three-four days a week, one to two days a week, one-two days per month, do not exercise regularly or cannot exercise due to my condition. Based on national recommendations, the level of physical activity was categorised as healthy if the patient engaged in physical exercise for 30 min at a moderate level for 5 days or more per week [16]. The national recommendations for physical activity state that people should also engage in physical activity that is of vigorous-intensity [16]. However, this was not included as it is not possible to report this in DANBIO.

Waistline was measured midway between the lower rib margin and iliac crest 2 cm above the umbilicus [17]. Waistline was categorised according to the International Diabetes Federation and circumference cut-points are gender specific [18]. Waistline was categorised as low risk (< 80 cm), intermediate risk (80–88 cm), and increased risk (> 88 cm) for women, and low risk (< 94 cm), intermediate risk (94–102 cm), and increased risk (> 102 cm) for men [18, 19]. Waistlines ≥ 80 cm for women and ≥ 94 cm for men, were categorized as unhealthy.

The patient’s BMI (kg/m^2)^ was categorised as underweight or normal weight (< 25 kg/m^2^), overweight (≥ 25 and < 30 kg/m^2^), or obese (≥ 30 kg/m^2^), and a BMI ≥ 25 kg/m2 was categorised as unhealthy.

#### Measures of disease impact

Disease activity measured by the Disease Activity Score C-reactive protein (DAS28-CRP) was not recorded during the screening consultation as this would require additional time and blood test. Still, inflammatory activity bears great importance for CV risk in patients with RA [3, 20]. To obtain this information, data were drawn from the most recent outpatient visit at the hospital, with a limit of three months before or after the screening consultation [10]. In addition, health-related quality of life measured by the EurolQol-5 Dimensions, 5 levels (EQ-5D-5L; 0–1, worst to best) [21], physical disability measured by the Health Assessment Questionnaire (HAQ; 0–3, best to worst) [22], pain, fatigue, and patient global assessment (PatGA) measured by visual analogue scales (VAS; 0–100, best to worst) [23], and data on comorbidities, were drawn from the same visit in DANBIO as DAS28-CRP. The recorded comorbidities encompassed known diabetes mellitus and CVD. CVD included hypertension, hypercholesterolemia, angina pectoris, myocardial infarction, stroke, vasoconstriction of the legs, and ‘other CVDs’.

### Statistical analysis

Descriptive data are presented with mean and standard deviation (SD) or median interquartile range (IQR), number and percentage (%), depending on the distribution of data. The age of the patients who were not invited to a screening consultation was calculated based on their first ever visit to the hospital. If their first visit was before September 2011, the participant’s age was calculated based on age in September 2011. Data for a first visit to the hospital were missing for 614 (28%) patients. Their age was calculated based on their birth year and the year of data retrieval (2021).

Differences between the two groups (< 5% vs. ≥ 5% risk for CVD) were analysed by univariate and multivariate logistic regression analyses. The explanatory variables were CV risk factors not included in the SCORE: triglycerides, blood glucose, lifestyle factors and measures of disease impact (pain, fatigue, PatGa, HAQ and EQ-5D-5L). The logistic regression analyses were performed fulfilling the one in ten rule to avoid overfitting [24], where the strongest predictors from the univariate analyses were included in the multivariate analyses. Waistline is considered more precise than BMI to predict CV risk, which is why BMI was excluded from the logistic regression analyses and from the calculation of the combined number of unhealthy lifestyle factors [17]. In case of missing data, this was assumed to be missing at random and therefore multiple imputation was conducted by way of Markov Chain Monte Carlo for continuous variables. The normality assumption of deviance residuals was assessed through QQ plots as the model control procedure. Furthermore, an evaluation of the final multivariate model’s performance was conducted by employing both discrimination and calibration metrics. The discrimination aspect was assessed using the C-statistic, also known as the Area Under the ROC Curve (AUC). Additionally, the Hosmer-Lemeshow goodness-of-fit test was employed to evaluate the model's calibration.

Differences between patients with high to very high CV risk who accepted vs. declined a follow-up screening were explored regarding age, gender, disease duration, disease activity, the combined number of unhealthy lifestyle factors and measures of disease impact (pain, fatigue, PatGa, HAQ and EQ-5D-5L), using the Wilcoxon rank sum test or chi-squared test. No imputation of missing values was used in the analyses, and the number of patients with available data for each variable is provided.

The statistical analyses were performed using STATA Version 17 (StataCorp, College Station, TX). The significance level was set to 5%. No correction for multiple testing was used. The planned statistical analyses were described in a statistical analysis plan before data were retrieved.

## Results

**Fig. 1:**
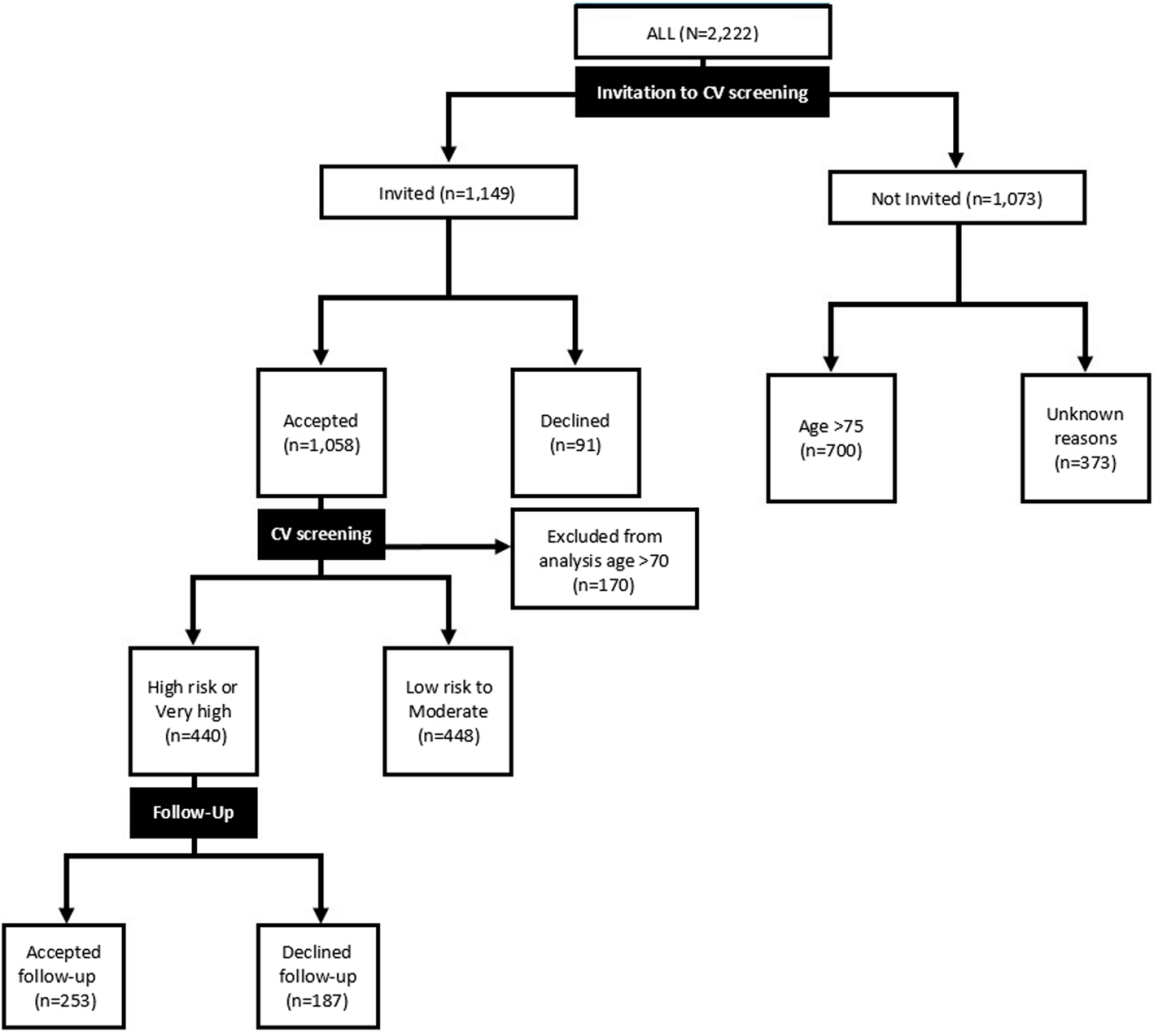
Flowchart of the cohort of the 2,222 outpatients diagnosed with RA connected to the Danish Hospital for Rheumatic Diseases between September 2011 and August 2021 and their participation in CV screening consultations

Between September 2011 and August 2021, 2,222 patients diagnosed with RA were connected to the outpatient department at the hospital, see Fig. 1. In total 1,522 of the outpatients with RA were under 75 years old and eligible to be invited for a CV screening consultation. However, 373 (25%) of the patients below 75 years with RA were not invited for a screening consultation, which was most likely due to an IT failure, leaving 1,149 invited patients (Fig. 1).

### Participation in CV screening consultations

Out of the 1,149 patients invited, 1,058 (92%) accepted and 91 (8%) declined participation (Fig. 1). Demographic and disease-related characteristics of the invited patients are shown in Table 1. The patients who declined an invitation to a screening consultation were more often men compared to the patients who accepted the invitation (*p* = *0.037*). Positive anti-cyclic citrullinated peptide were more common in patients who accepted the invitation compared to those who declined (*p* = *0.034*). Furthermore, patients who declined the invitation to a screening consultation had worse disease activity compared to those who accepted an invitation to a screening consultation (Table 1) (*p* = *0.017*).

**Table 1:** Demographic and disease characteristics of the patients who were invited to a CV screening consultation

**Variables**	***n***	**Accepted invitation to screening** ***n* = 1,058**	**Declined invitation to screening** ***n* = 91**	***p*-value**
Age, years	1,149	60.5 (9.30)	59.4 (14.35)	0.291
Gender, men	344	308 (29%)	36 (40%)	**0.037**
Disease duration, years	1,128	9.37 (9.91)	10.4 (9.24)	0.350
Positive RF	296	178 (64%)	7 (41%)	0.061
Positive Anti-CCP	469	279 (64%)	15 (46%)	**0.034**
DAS28-CRP	942	2.63 (1.20)	3.03 (1.55)	**0.017**
Medical treatment	1,108			
No DMARD or CS		1 (1%)	-	-
cDMARD ± CS		781 (76%)	67 (80%)	-
bDMARD ± CS		166 (16%)	9 (10%)	-
Only CS		76 (7%)	8 (10%)	0.392
Known CVD	1,054	462 (44%)	-	-
Known DM,	1,047	77 (7%)	-	-

Data presented as mean (standard deviation) or n (%).Wilcoxon’s rank-sum test was employed for continuous variables; chi-squared test for categorical variables. *P*-values < 0.05 considered statistically significant are presented in bold. *n* = number of patients with available data for each variable is provided

*RF* rheumatoid factor, *Anti-CCP* anti-cyclic citrullinated peptide, *DAS28-CRP* disease activity score in 28 joints–CRP and patient global assessment score (0–10), *DMARD* disease-modifying antirheumatic drug, *cDMARD* conventional disease-modifying antirheumatic drugs (with or without corticosteroids), *bDMARD* biological disease-modifying antirheumatic drugs (with or without corticosteroids), *CS* corticosteroids, *CVD* cardiovascular diseases, *DM* Diabetes Mellitus

### Differences in CV risk factors in patients with low to moderate vs. high to very high risk

As a part of the clinical practice and based on the EULAR recommendations [3], all patients under the age of 75 years are invited for CV screening consultations. However, SCORE system is only valid for people under the age of 70 and the SCORE risk chart will underestimate the risk of patients over 70 years old [4]. Of the 1,058 patients who accepted the invitation, 170 were over 70 years old, and these patients were excluded from further analyses as we do not have a risk SCORE. The underestimation of risk also applies to patients with known CVD or DM [4]. A total of 354 (40%) patients reported being diagnosed with CVD or DM and these patients were coded as having high to very high risk SCORE.

A mSCORE was calculated for 888 patients and they were dichotomised into low to moderate risk (mSCORE < 5%) or high to very high risk (mSCORE ≥ 5%) (Table 2). In total, 448 (51%) patients had low to moderate risk, and 440 (49%) had high to very high risk. Patients with high to very high risk had longer disease duration than those with low to moderate risk (*p* = *0.013*). Furthermore, patients with high to very high risk had significantly higher level of triglycerides, and fasting glucose or HbA1C, compared to patients with low to moderate risk (*p* < *0.001*). There was also a significant difference in terms of mSCORE in patients with RA and the calculated SCORE (*p* < *0.001*). 31 (7%) of the patients with RA would have been classified as low to moderate risk if the SCORE had not been multiplied with 1.5. In terms of unhealthy lifestyle factors, patients with high to very high risk reported higher waist circumference and higher BMI than those with low to moderate risk. This included high waistline, ≥ 80 cm for women and ≥ 94 cm for men, and BMI ≥ 30. The presence of two or more unhealthy lifestyle factors were more frequent in patients with high to very high risk than in patients with low to moderate risk *(60% vs. 53%, p* = *0.026).* In addition, patients with high to very high risk more often declined the invitation to a follow-up screening than those with low to moderate risk *(p* < *0.001*) (Table 2).

**Table 2:** The spread of cardiovascular risk factors for patients ≤ 70 years who participated in at least one CV screening (*n* = 888)

**Variables**		***n***	**mSCORE < 5%** ***n* = 448 (51%)**	**mSCORE ≥ 5%** ***n* = 440 (49%)**	***p*-value**
Age, years		888	54.0 (48.0–61.0)	64.0 (58.0–67.0)	** < 0.001**
Gender, Male		251	90 (20%)	161 (37%)	** < 0.001**
Gender, Female		637	358 (80%)	279 (63%)	** < 0.001**
Disease duration, years		875	5.0 (2.0–12.0)	7.0 (2.0–15.0)	**0.013**
Triglycerides (mM/L)		886	1.0 (0.8–1.4)	1.2 (0.9–1.7)	** < 0.001**
HbA1c (mM/L)		409	34.7 (21–45)	38.0 (19–95)	** < 0.001**
Fasting glucose		433	5.3 (3.2–14.6)	5.6 (4.1–10.1)	** < 0.001**
SCORE < 5%		479	448 (100%)	31 (7%)	**-**
SCORE ≥ 5%		409	0	409 (93%)	** < 0.001**
Adherence to a follow-up screening consultation					
Accepted		574	321 (72%)	253 (57%)	-
Declined		314	127 (28%)	187 (43%)	** < 0.001**
Unhealthy lifestyle factors					
Alcohol^a^					
Below recommended limits		784	396 (87%)	388 (88%)	-
Above recommended limits		104	52 (13%)	52 (12%)	0.922
Physical activity (≥ 30 min/day)					
0–2 days per month		297	139 (31%)	158 (36%)	-
1–4 days per week		337	181 (40%)	156 (36%)	-
5 or more days per week		253	128 (29%)	125 (28%)	0.222
Waistline^b^					
	Low risk	249	154 (35%)	95 (22%)	
Intermediate risk		223	123 (28%)	100 (23%)	
Increased risk		406	167 (37%)	239 (55%)	** < 0.001**
BMI, kg/m^2^					
	BMI, kg/m^2^ < 25.0	273	165 (41%)	108 (27%)	
	BMI, kg/m^2^ 25.0–29.9	280	143 (35%)	137 (34%)	
	BMI, kg/m^2^ ≥ 30	252	98 (24%)	154 (39%)	** < 0.001**
Combined number of unhealthy lifestyle factors^c^					
0		73	44 (10%)	29 (7%)	-
1		308	165 (37%)	143 (33%)	-
2		439	211 (48%)	228 (53%)	-
3		58	24 (5%)	34 (8%)	0.074
Combined number of unhealthy lifestyle factors (0–1 vs. ≥ 2)^c^					
	0–1	381	209 (47%)	172 (40%)	-
	≥ 2	497	235 (53%)	262 (60%)	**0.026**

Data presented as median (IQR) or n (%). Wilcoxon’s rank-sum test was employed for continuous variables; chi-squared test for categorical variables. *P*-values < 0.05 considered statistically significant are presented in bold. n = number of patients with available data for each variable is provided

*CVD* cardiovascular disease, *DM* diabetes mellitus, *BMI* body mass index calculated as weight (kg)/squared height (m2), *mSCORE* modified Systematic Coronary Risk Evaluation, *SCORE* Systematic Coronary Risk Evaluation

^a^Max. 7 units/week for women and 14 for men

^b^Women: low risk (< 80 cm), intermediate risk (80–88 cm), increased risk (> 88 cm) Men: low risk (< 94 cm), intermediate risk (94–102 cm), increased risk (> 102 cm)

^c^Combination of alcohol, physical activity, and waistline

### Differences between patients with low to moderate vs. high to very high risk

The results from the univariate and the multivariate logistic regression analyses on differences between patients with low to moderate vs. high to very high risk are shown in Table 3. In the crude analyses, patients with high to very high risk had significantly longer disease duration (OR 1.017, 95% CI 1.002-1.032), higher level of triglycerides (OR 1.834, 95% CI 1.475-2.280), higher blood sugar (OR 1.046, 95% CI 1.02-1.070), worse pain (OR 1.006, 95% CI 1.001-1.012), and worse HAQ (OR 1.305, 95% CI 1.057-1.612) compared to patients with low to moderate risk (Table 3).

**Table 3:** Differences between patients SCORE < 5% vs. mSCORE 5%, (*n* = 888)

**Variables**		**Crude OR**		**Multivariate analysis** **Adjusted OR**	
		**(CI 95%)**^a^	***p*-value**	**(CI 95%)**^a^	***p*-value**
Disease duration, years		1.017 (1.002–1.032)	**0.019**	1.028 (1.008–1.048)	**0.005**
Triglycerides		1.834 (1.475–2.280)	** < 0.001**	1.457 (1.126–1.886)	**0.004**
HbAlc		1.046 (1.020–1.070)	** < 0.001**	1.033 (0.999–1.066)	0.055
Unhealthy lifestyle factors					
Alcohol above national limits	Women ≥ 7	1.242 (0.732–2.107)	0.421	–	–
	Men ≥ 14	0.838 (0.408–1.718)	0.630	–	–
Physical activity	0–2 days per month	1	Ref	1	Ref
	1–4 days per week	0.758 (0.555–1.036)	0.083	0.909 (0.605–1.366)	0.647
5 or more days per week		0.859 (0.614–1.201)	0.375	1.020 (0.654–1.591)	0.928
High waist circumference	Women ≥ 80 cm	0.712 (0.432–1.174)	0.183	0.585 (0.322–1.064)	0.079
	Men ≥ 94 cm	1.451 (0.829–2.540)	0.192	1.001 (0.509–1.971)	0.996
Measure of disease impact					
Pain		1.006 (1.001–1.012)	**0.022**	1.027 (1.011–1.045)	**0.001**
Fatigue		1.000 (0.995–1.005)	0.884	0.994 (0.982–1.001)	0.321
PatGA		1.001 (0.996–1.061)	0.575	0.978 (0.962–0.996)	**0.014**
HAQ		1.305 (1.057–1.612)	**0.013**	1.33 (0.91–1.96)	0.137
EQ-5D-5L		0.39 (0.311–0.531)	0.531	0.722 (0.195–2.669)	0.640

^a^Logistic regression analyses

Data presented as odds ratio (OR) and 95% confidence intervals

Pain (0–100, best to worst); Fatigue (0–100, best to worst)

*PatGa* patient global assessment (0–100, best to worst); pain (0–100, best to worst), *HAQ* Health Assessment Questionnaire (0–3, best to worst), *EQ-5D-5L* Five level Euroqol Five Dimensions (0–1, worst to best)

Statistically significant p-values are in bold

In the final multivariate analysis, a decision was made to exclude alcohol above national limits. This decision was justified by the lack of statistical significance observed in the univariate analysis. In the multivariate model, significant differences were found in terms of longer disease duration (OR 1.028, 95% CI 1.008–1.048), higher levels of triglycerides (OR 1.457, 95% CI 1.126–1.886), worse pain (OR 1.027, 95% CI 1.011–1.045), and better global health (OR 0.978, 95% CI 0.962–0.996) in patients with high to very high risk compared to patients with low to moderate risk.

The final multivariate logistic regression model demonstrated acceptable calibration, as evidenced by a non-significant *p*-value in the Hosmer–Lemeshow goodness of fit test (*p* = *0.691*). The C-statistic, with its associated confidence interval, suggests that the model has reasonable discriminatory power, 0.712 (95% confidence interval: 0.642–0.801).

We conducted a sensitivity analysis, excluding patients with known CVD or DM (*n* = *71*), but the overall results from the multivariate analysis did not change.

### Adherence to follow-up screening in patients with high to very high risk

Of the 440 patients with high to very high risk, 58% accepted a follow-up screening consultation. Patients who declined a follow-up screening consultation were older and had a shorter disease duration compared to those who accepted a follow up screening consultation (Table 4). There was no difference in the combined number of unhealthy lifestyle factors between those who accepted versus declined the invitation to a follow-up screening in the group with high or very high risk. A sensitivity analysis excluding patients with known CVD or DM did not identify significant differences between the two groups.

**Table 4:** Differences between patients with high risk who accepted vs. declined a follow-up screening (*n* = 440)

**Variables**	***n***	**Accepted** ***n* = 253 (58%)**	**Declined** ***n* = 187 (42%)**	***p*-value**
Age, years	440	63.0 (57.0–67.0)	65.0 (59.0–68.0)	**0.016**
Gender, male	161	93 (58%)	68 (42%)	0.932
DAS28-CRP	364	2.4 (1.7–3.1)	2.6 (1.8–3.7)	0.090
Disease duration, years	440	8.0 (3.0–15.0)	5.5 (2.0–13.0)	**0.006**
Unhealthy lifestyle factors				
Alcohol number of units (*n* = *440*)				
Women ≥ 7	25	15 (9%)	10 (8%)	0.779
Men ≥ 14	27	19 (20%)	8 (12%)	0.146
Physical activity (*n* = *339*)				
0–2 days per month	158	86 (34%)	72 (39%)	-
1–4 days per week	156	89 (35%)	67 (36%)	-
5 or more days per week	125	78 (31%)	47 (25%)	0.400
Waistline (*n* = 434)				
Women ≥ 80 cm	223	126 (79%)	97 (82%)	0.445
Men ≥ 94 cm	116	68 (73%)	48 (71%)	0.919
Combined number of unhealthy lifestyle factors^a^ (*n* = 434)				
0–1	172	102 (40%)	70 (40%)	-
≥ 2	262	150 (60%)	112 (60%)	0.670
Measures of disease impact				
Pain (0–100)	420	27.0 (11.0–52.0)	33.5 (11.0–57.0)	0.360
Fatigue (0–100)	400	32.5 (15.0–60.0)	41.5 (15.0–65.0)	0.141
PatGA (0–100)	421	26.0 (11.0–54.0)	36.0 (11.0–63.0)	0.086
HAQ (0–3)	408	0.5 (0.1–1.1)	0.8 (0.3–1.1)	0.068
EQ-5D-5L (0–1)	95	0.8 (0.7–0.9)	0.8 (0.7–0.8)	0.370

Data presented as median (IQR) or n (%). Wilcoxon’s rank-sum test was employed for continuous variables; chi-squared test for categorical variables. P-values < 0.05 considered statistically significant are presented in bold. n = number of patients with available data for each variable is provided

*DAS28–CRP* disease activity score in 28 joints–CRP and patient global assessment score (0–100); Pain (0–100, best to worst); Fatigue (0–100, best to worst), *PatGa* patient global assessment (0–100, best to worst); pain (0–100, best to worst), *HAQ* Health Assessment Questionnaire (0–3, best to worst), *EQ-5D-5L* Five level Euroqol Five Dimensions (0–1, worst to best)

^a^Combination of alcohol, physical activity and waistline

## Discussion

Of the 1,522 eligible outpatients with RA, (75%), under the age of 75 years, connected to the outpatient clinic at the Danish Hospital for Rheumatic Diseases, were invited to a CV screening consultation, and 91% of the invited patients accepted the invitation. Patients with high to very high risk for CV death were more often male and surprisingly, they reported a better global health compared to the patients with low to moderate risk. Furthermore, they also reported longer disease duration, higher levels of triglycerides, and more pain. In total, 58% of the patients with high to very high risk participated in a follow-up screening consultation. The patients who declined follow-up were older and had a shorter disease duration than those who accepted.

The 8% of the invited patients who declined participation were more often men with a worse disease activity score compared to those who accepted a screening invitation. In a previous study, we found that approximately 10% declined the invitation in the first year after implementation of systematic CV screening, and the reasons given for this were severe disability, comorbidities, difficulties with transportation, or work-related issues [13]. Two Canadian studies found that participation in lipid screening did not differ and participation in glucose screening was only slightly increased among patients with RA compared to the general population [25, 26]. Thus, their participation does not seem to reflect their increased risk for CVD. In Denmark, patients with known DM are monitored by their general practitioner (GP) or in a diabetes clinic at a hospital where their CV risk is also assessed, and this may be the reason why some patients with DM and RA may decline such invitations.

In both the crude analyses and the multivariate model, we found that patients with high to very high risk had significantly higher levels of triglycerides than those with low to moderate risk, but the difference was small. A relationship between total-/HDL-cholesterol and triglycerides has been found [27]. As the total-/HDL-cholesterol ratio is included in the mSCORE, this may explain the small but significant difference between the two groups. Similar to this, a correlation between HbA1C and lipid-profile was found in patients with diabetes [28].

Although a yearly follow-up screening is recommended for patients with high to very high risk, [3], only six out of ten among the 440 patients with high to very high risk accepted the follow-up invitation and a larger proportion declined the follow-up invitation than among those with low to moderate risk. In a previous study we found that patients with RA did not always understand that they had high CV risk [27], and this may help explain why some with high risk declined the follow-up invitation. It is important to acknowledge that participation in CV screening does not per se change unhealthy lifestyle factors. A study from the Netherlands stated that there is a need for immediate follow-up for patients with RA and high risk for CVD after participation in CV screening to support lifestyle changes and/ or transfer to their GP. In the Dutch study, only 24% of patients with high CV risk followed the advice for a follow-up with their GP, even though both parties received information regarding the patients CV risk [11]. However, in a register based study including all patients with inflammatory arthritis from the outpatient department at the Danish Hospital for Rheumatic Diseases, 75% of the patients with high risk visited their GP during the first six weeks post-screening [29]. In a previous study, we found that some felt anxious when invited for a CV screening consultation, but they were glad that they could do something about their CV risk profile if i.e. glucose or HbA1C was high [30].

A large proportion of patients with RA have two or more unhealthy lifestyle factors and this is associated with CV death in the general population [7, 8, 10]. In this study, patients with high to very high risk more often reported two or more unhealthy lifestyle factors compared to those with low to moderate risk. No difference was found in the combined number of unhealthy lifestyle factors between those who accepted or declined an invitation for follow-up in patients with high to very high risk. Also, patients with high to very high risk reported higher waist circumference and higher BMI than those with low to moderate risk. A Dutch study found that the prevalence of smokers was lower among patients with RA who attended CV prevention than in the general RA population [31]. This may indicate that some patients decline participation in CV screening due to their unhealthy lifestyle habits. Some patients with RA may not be interested in engaging in a discussion on unhealthy lifestyle habits with a health professional [32]. In addition, male gender is associated with a higher risk of CVD in the general population [17], and this is also applicable in this study where a large proportion of the RA patients with a high to very high risk for CVD were men. Previous findings also showed that two or more unhealthy lifestyle factors were more common in men, supporting the higher risk of CVD [10]. This indicates that health professionals also need to focus on the gender differences in CVD risk, and especially on how to support men to implement lifestyle changes. The reasons for high risk patient’s adherence or non-adherence to the CV follow-up screening are not known, and there is limited knowledge on the patients’ perception and experience from participating in a CV screening. Thus, the patients’ perspective on participation, barriers, and facilitators for adherence need further exploration.

### Strengths and limitations

One of the strengths of the current study is the systematic collection of data, and the large proportion of patients who participated in a CV screening consultation. Furthermore, all data were reported in a national registry. The database was updated in 2021 and it now appears that the 373 patients who had not previously been invited for a screening, have now been invited. This indicates a previous systematic IT-failure and a need to monitor implementation of new activities closely. The study also has some limitations. Unfortunately, reasons for declining the invitation to CV screening and follow-up was not registered as the offer is part of clinical practice. Furthermore, information regarding medical treatment in relation to CV risk factors, such as lipid lowering, and antihypertensive drugs could have added valuable information regarding the patients’ 10-year risk for CV death, but this information was not available in the registers. Treatment with high doses of steroids has also been shown to negatively affect the patient’s CV risk compared to those who are not treated with steroids [33]. Unfortunately, we do not have valid data for the patient’s use of steroids. At the hospital, steroids are primarily used to treat inflammation until initiation or switch of a treatment with DMARDs is effective. Very few patients receive steroids on a daily basis. Another limitation is the lack of information about other comorbidities, such as chronic kidney disease and lung diseases. Only valid information about present CVD and DM were available. The sample size in the sensitivity analyses is small, which infer a risk for a type 2 error. Thus, a difference between the groups, when excluding patients with known comorbidities, cannot be ruled out. Furthermore, data regarding measures of disease impact was not from the same date as the CV screening consultation.

## Conclusion

In total, 75% of the outpatients under the age of 75 years old were invited to a CV screening consultation. Nine out of ten patients with RA accepted the first invitation to a CV screening consultation. The patients who declined the invitation were more often men and had worse disease activity. Patients with high to very high risk had longer disease duration, higher levels of triglycerides, and more pain, but reported better global health and there were more males than among those with low to moderate risk. Approximately six out of 10 of the patients with high to very high risk for CV death adhered to a follow-up screening consultation. The patients with high to very high risk who declined the invitation to follow-up screening were older and had shorter disease duration compared to those who accepted follow-up screening. Neither measures of disease impact nor lifestyle factors were associated with adherence. Further studies are needed to explore the patient’s motivation, barriers and facilitators for adherence or non-adherence to a follow-up CV screening consultation.

## Data Availability

The data used in this study is not publicly available due to legal and ethical reasons.

## Abbreviations

RA: Rheumatoid arthritis
CVD: Cardiovascular disease
EULAR: European Alliance of Associations for Rheumatology
CV: Cardiovascular
SCORE: Systematic Coronary Risk Evaluation
mSCORE: Modified Systematic Coronary Risk Evaluation
HDL: High density lipoprotein
LDL: Low-density lipoprotein
BMI: Body mass index
DM: Diabetes Mellitus
DMARD: Disease-modifying antirheumatic drug
CS: Corticosteroids
DAS28-CRP: Disease Activity Score C-reactive protein
EQ-5D-5L: EurolQol-5 Dimensions, 5 levels
HAQ: Health Assessment Questionnaire
PatGA: Patient global assessment
VAS: Visual analogue scales
SD: Standard deviation
IQR: Interquartile range
GP: General practitioner
